# Adverse stroke outcomes among patients with bipolar disorder

**DOI:** 10.1371/journal.pone.0213072

**Published:** 2019-03-04

**Authors:** Pao-Huan Chen, Yi-Wei Kao, Ben-Chang Shia, Herng-Ching Lin, Jiunn-Horng Kang

**Affiliations:** 1 Department of Psychiatry, Taipei Medical University Hospital, Taipei, Taiwan; 2 Department of Psychiatry, School of Medicine, College of Medicine, Taipei Medical University, Taipei, Taiwan; 3 Graduate Institute of Clinical Medicine, College of Medicine, Taipei Medical University, Taipei, Taiwan; 4 Big Data Research Center, Taipei Medical University, Taipei, Taiwan; 5 School of Health Care Administration, Taipei Medical University, Taipei, Taiwan; 6 Sleep Research Center, Taipei Medical University Hospital, Taipei, Taiwan; 7 Department of Physical Medicine and Rehabilitation, Taipei Medical University Hospital, Taipei, Taiwan; 8 Department of Physical Medicine and Rehabilitation, School of Medicine, College of Medicine, Taipei Medical University, Taipei, Taiwan; University of L’Aquila, ITALY

## Abstract

Failure to deliver the standard stroke care is suspected to be a potential reason for disproportionately high mortality among patients with co-morbid bipolar disorder (BD). Few studies have explored adverse outcomes and medical care costs concurrently (as a proxy for care intensity) among patients with BD admitted for stroke. Data for this nationwide population-based study were extracted from the Taiwan National Health Insurance Research Database, on 580 patients with BD hospitalized for stroke (the study group) and a comparison group consisting of randomly selected 1740 stroke patients without BD matched by propensity scores. Conditional logistic regression was used to estimate odds ratios (OR) for adverse in-hospital outcomes between study group and comparison group. We found that stroke patients with BD had significantly lower in-hospital mortality (3.28% vs. 5.63%), acute respiratory failure (2.59% vs. 5.57%), and use of mechanical ventilation (6.55% vs. 10.23%) than the comparison group. After adjusting for geographical location, urbanization level, monthly income, hypertension, diabetes, hyperlipidemia, and coronary heart disease, the odds of in-hospital mortality, acute respiratory failure, and use of mechanical ventilation in the BD group were 0.56 (95% CI: 0.34–0.92), 0.46 (95% CI: 0.26–0.80), and 0.63 (95% CI: 0.44–0.91), respectively. No differences were found in hospitalization costs and the length of hospital stay. With comparable hospitalization costs and length of hospital stay, we concluded that stroke patients with BD had lower in-hospital mortality and serious adverse events compared to stroke patients without BD.

## Introduction

Bipolar disorder (BD) is a serious mental illness causing a high degree of medical burden during the course of illness and with the aging process [1]. Notably, patients with BD are documented to have 2 to 4 times higher mortality and at least a 10-year reduction in life expectancy compared to the general population [1,2]. Furthermore, evidence suggests that the mortality gap has widened in recent decades [3]. Among the medical causes of excessive and premature mortality deaths among BD patients, cardiovascular diseases are recognized as the leading cause, reported in both Western and Eastern studies [4–7].

Stroke is a cardiovascular disease with a high risk of mortality and long-term disability [8,9]. General population studies have shown that obesity, diabetes, dyslipidemia, hypertension, cigarette smoking, and alcohol abuse are the major risk factors for stroke [10]. Studies also suggest that patients with BD are more susceptible to develop or have the stroke risk factors than the general population [11–15]. One proposed reason is suspected to be suboptimal medical care of metabolic and vascular diseases in patients with BD [16–20].

Studies, worldwide, have shown that patients with BD experience suboptimal treatment for stroke [7,15,21]. Stroke care guidelines recommend specific and detailed medical care plans to limit complications and improve stroke outcomes [22]. Although patients with BD are about twice as likely to develop stroke as the general population [1,5,7,15,23,24], in-patient admission rates for cardiovascular care are only slightly higher in patients with BD [7,15]. A few studies have reported that patients with BD may suffer from poor outcomes following a stroke [7,25]. Failure to deliver adequate medical care to these patients is postulated to contribute to their poorer outcomes following stroke and cardiovascular diseases. However, there are no studies examining concurrently, the medical care costs and in-hospital adverse outcomes of patients with BD admitted for a stroke. In this study, we used a nationwide, population-based database to investigate both adverse outcomes and acute hospitalization costs patients with stroke. We hypothesized that BD could significantly affect the in-hospital outcomes of stroke. We investigated total medical cost as a proxy for care intensity comparing patients with BD with the comparison group to evaluate the role of potentially differential care provision in the documented adverse outcomes of patients with BD with stroke. Such an analysis is essential to understand the patterns of care provided to patients with BD when they experience non-psychiatric medical conditions.

## Methods

### Database

Data for the study were drawn from the Taiwan National Health Insurance Research Database (NHIRD). The NHIRD maintains registration files and medical claims data for approximately 99% of the Taiwanese population (n = 23 million) provided care under Taiwan's National Health Insurance (NHI) program. The NHI is a single-payer program initiated in 1995. It provides comprehensive and accessible medical care for all residents. Enrollees can choose to visit any physician or hospital among the NHI-contracted facilities throughout Taiwan. The NHIRD has enabled a large number of longitudinal studies following specific medical services of the Taiwan population since the beginning of the NHI program. This study was exempt from full review by the institutional review board of Taipei Medical University (TMU-JIRB201708042), because NHIRD releases de-identified and encrypted secondary data to the public for academic research.

### Study sample

This is a cross-sectional study, starting with 295,677 patients hospitalized with a principal diagnosis of stroke (ICD-9-CM codes 430–437) between January 1, 2010 and December 31, 2014. The first claim date with a diagnosis of stroke was identified as the index date. For patients hospitalized for stroke more than once during the study period, their first hospitalization was defined as the index hospitalization. We excluded 1,849 sampled patients who were aged <18 years because of very low prevalence of stroke in this age group. Among the remaining 293,828 adult patients with stroke, we identified 580 patients who had ever received a diagnosis of BD (ICD-9-CM code ICD-9-CM code of 296.0, 296.4, 296.5, 296.6, 296.7, 296.80 or 296.89) from the Registry of Catastrophic Illness Patient Database as the study group. In Taiwan, patients with BD qualify to apply for a catastrophic illness card that can decrease the financial burden of patients with serious illness. The application of a catastrophic illness card must be signed by a board-certificated psychiatrist after the diagnosis. Furthermore, the NHI Bureau reviews the medical records to verify the diagnosis. Therefore, the selection of patients from the Registry for Catastrophic Illness Patient Database greatly enhances the validity of these disease diagnoses.

We identified a comparison group from the remaining stroke patients. We first excluded patients who had ever received a diagnosis of schizophrenia or BD. We then calculated a propensity score for each patient with BD in the study. Propensity scores have been widely used to balance the demographic and health status characteristics, which were distributed unequally between patients with BD and comparison group. In this study, propensity score development starts with identification of the factors that could influence BD including patient demographics and comorbidities. Patient demographics and comorbidities were entered into a multivariable logistic regression model to calculate the probability of being diagnosed with BD. Patient demographics included age, sex, monthly income, geographic location (Northern, Central, Southern and Eastern) and urbanization level of the patient’s residence. Comorbidities included hypertension, hyperlipidemia, diabetes and coronary heart disease. After the development and assessment of propensity scores, we matched sample of BD patients and comparison patients who have equal or similar propensity scores. The final sample consisted of 2320 patients, 580 study patients and 1740 comparison patients (three for every patient with BD).

### Outcome measures

Adverse outcomes of interest were in-hospital mortality, pneumonia (ICD-9-CM codes 480–483.8, 485–486, and 487.0), urinary tract infections (UTIs) (ICD-9-CM codes of 590, 590.0–590.9, 599.0, 595.0 or 595.9), acute respiratory failure (ICD-9-CM code 518.81), and the use of mechanical ventilation (ICD-9-CM procedure codes 967 and 967.0–967.2). In-hospital mortality was defined as ‘death of a patient at any time after admission if the patient did not leave the hospital alive’. We also studied hospitalization variables, including length of stay and hospitalization costs, comparing stroke patients with BD and without BD. Hospitalization costs were defined as the total monetary amount of medical benefits claimed in the index hospitalization.

### Statistical analysis

All statistical analyses were performed using SAS statistical software (SAS System for Windows, vers. 9.2, SAS Institute, Cary, NC). Chi-square tests were used to compare differences in sex, monthly income, geographical location (northern, central, eastern, and southern Taiwan), urbanization level (seven levels, 1 most urbanized and 7 least urbanized), and comorbidities (hypertension, diabetes, hyperlipidemia, and coronary heart disease). Student’s t-test was used to compare age composition of the two groups. We used conditional logistic regressions to estimate odds ratios (ORs) and 95% confidence intervals (CIs) for adverse outcomes between the study group and comparison group. To study hospitalization variables, we performed multivariate regression analyses using log-transformed values of length of stay and hospitalization costs. A p value of <0.05 was used to determine statistical significance.

## Results

Table 1 shows the demographic characteristics and comorbidities of the study group and comparison group. Given the use of propensity-score matching, the two groups were similar on most characteristics: mean ages of the study group and comparison group were 62.3±14.8 and 62.0±14.7 years, respectively (*p* = 0.63), with no difference in sex, urbanization level, hypertension, hyperlipidemia, diabetes, and coronary heart disease distribution. However, the groups differed on geographic region (*p* = 0.007) and monthly income (*p* = 0.03). Furthermore, of the 580 patient with BD, 26.8%, 24.0%, 16.5%, 13.8% and 18.9% were diagnosed with bipolar affective disorder, depressed (ICD-9-CM code 296.5), bipolar affective disorder, manic (ICD-9-CM code 296.4), bipolar affective disorder, mixed (ICD-9-CM code 296.6) manic-depressive psychosis, unspecified (ICD-9-CM code 296.80) and others (ICD-9-CM codes 296.0, 296.7 or 296.89), respectively.

**Table 1:** Demographic characteristics and comorbidities of stroke patients with and those without bipolar disorder (*N* = 2,320).

Variable	Patients with bipolar disorder *N* = 580		Comparison patients *N* = 1740		*p* value
	Total no.	Column %	Total no.	Column %	
Age (years), mean (SD)	62.3±14.8		62.0±14.7		0.63
Male	286	49.3	897	51.6	0.35
Geographic region					0.007
Northern	243	41.9	753	43.3	
Central	171	29.5	406	23.3	
Southern	146	25.2	534	30.7	
Eastern	20	3.5	47	2.7	
Urbanization level					0.94
1	149	25.7	424	24.4	
2	168	29.0	481	27.6	
3	90	15.5	281	16.2	
4	86	14.8	265	15.2	
5	15	2.6	56	3.2	
6	26	4.5	78	4.5	
7	46	7.9	155	8.9	
Monthly income (US$)					0.029
$1~530	358	61.7	1131	65.0	
$530~829	165	28.5	495	28.5	
≥$830	57	9.8	114	6.6	
Comorbidities					
Hypertension	458	79.0	1399	80.4	0.45
Hyperlipidemia	324	55.9	971	55.8	0.98
Diabetes	100	17.2	244	14.0	0.06
Coronary heart disease	211	36.4	655	37.6	0.59
Anxiety disorder	402	69.3	444	25.5	<0.001
ADHD/hyperkinetic disorder	3	0.5	—	—	—
Dementia	149	25.7	212	12.2	<0.001
Substance-related disorder	118	20.3	99	5.7	<0.001

Table 2 presents the occurrence rate of the adverse outcomes of interest and hospitalization variables among patients with and without BD. Stroke patients with BD had significantly lower in-hospital mortality (3.28% vs. 5.63%), and event rates for acute respiratory failure (2.59% vs. 5.57%), and use of mechanical ventilation (6.55% vs. 10.23%). There were no statistically significant differences in pneumonia, UTIs, length of stay, and medical costs.

**Table 2:** Occurrence of adverse events and profile of hospitalization variables among stroke patients, stratified by the presence of bipolar disorder.

Outcomes	Total sample *N* = 2320	Patients with bipolar disorder *N* = 580	Comparison patients *N* = 1740	*p* value
	No. (%) or mean ± SD			
Adverse outcomes				
In-hospital mortality	117	19 (3.28)	98 (5.63)	0.025
Pneumonia	152	30 (5.17)	122 (7.01)	0.12
Urinary tract infections	200	60 (10.34)	140 (8.05)	0.09
Acute respiratory failure	112	15 (2.59)	97 (5.57)	0.004
Mechanical ventilation	216	38 (6.55)	178 (10.23)	0.008
Hospitalization outcomes				
Length of stay (days)	12.47±16.41	11.87±16.11	12.67±16.51	0.27
Medical costs (US$)	2182±3321	2012±2300	2252±3336	0.25

Note: SD, standard deviation.

Adjusted associations of BD with the adverse outcomes, and hospitalization variables are presented in Table 3. Logistic regressions showed that after adjusting for the demographic and comorbidity covariates ORs for in-hospital mortality, acute respiratory failure, and use of mechanical ventilation for patients with BD were 0.56 (95% CI: 0.34–0.92), 0.46 (95% CI: 0.26–0.80), and 0.63 (95% CI: 0.44–0.91), respectively, relative to those without BD. BD was negatively associated with in-hospital mortality, acute respiratory failure, and use of mechanical ventilation among stroke patients.

**Table 3:** Adjusted relationships between the occurrence of bipolar disorder, adverse outcomes, and hospitalization outcomes.

Outcome variables	Patients with bipolar disorder vs. Comparison patients
	Adjusted odds ratio ^a^ (95% CI)
Adverse events	
In-hospital mortality	0.56* (0.34–0.92)
Pneumonia	0.75 (0.50–1.13)
Urinary tract infections	1.36 (0.99–1.88)
Acute respiratory failure	0.46* (0.26–0.80)
Mechanical ventilation	0.63* (0.44–0.91)
Hospitalization variables	Parameter estimate (SE)
Log (length of stay)	-0.003 (0.003)
Log (medical costs) (US$)	-0.082 (0.084)

Notes: SE, standard error.

^a^ Adjustments were made for patient’s geographical location, urbanization level, monthly income, hypertension, diabetes, hyperlipidemia, and coronary heart disease

* p<0.05.

## Discussion

The present study may be the first to investigate in-hospital mortality and adverse event rates in stroke patients with BD. With comparable total hospitalization costs and length of stay, we found that stroke patients with BD actually had lower rates of in-hospital mortality, acute respiratory failure, and mechanical ventilation use than comparison patients without BD. On previous study had shown that the annual hospitalization cost of patients with BD for non-psychiatric causes was higher than patients without BD in Taiwan [15]. However, prior studies also revealed that admission rates for stroke among patients with BD were similar to those of the general population despite their higher risk of suffering a stroke [15, 21]. Taken together, the previous research findings suggest that the poor outcomes of stroke in patients with BD may result from the inadequate stroke care. In the present study, we have observed that, with the comparable hospitalization costs and length of hospital stay, stroke patients with BD had lower in-hospital mortality and serious adverse events compared to stroke patients without BD. The results suggest that the high mortality rates of stroke among patients with BD may possibly be reduced by optimal stroke care. Therefore, further studies utilizing the database from other health care systems are warranted to validate our present observations.

Contrary to the literature [7,21,25], we found that stroke patients with BD had lower in-hospital mortality and adverse event rates compared to the comparison group. Schoepf et al. found patients with BD were at an increased risk of in-hospital mortality following a stroke compared to the control group among their patient sample drawn from general hospitals [25]. In particular, the mean age of patients with BD at baseline in their study were 47.3±0.2 years and the duration of follow-up was 1814 days, much younger than our sample (62.3±14.8 years). The age discrepancy between the present study and previous studies remains inexplicable and may reflect differences in sampling methodology. Age is a critical factor in the management of patients with BD. Recently, the International Society for Bipolar Disorders Task Force proposed the age of 50 years as the demarcation to define older-age BD because of the higher medical burden and shorter lifespan in patients with BD [26]. Evidence suggests that patients with BD have an increased risk of cardiovascular disease from the fourth decade [7].

Prior studies suggested that patients with BD usually received suboptimal medical care for non-psychiatric diseases compared to the general population [16,18,19,20]. This suggests another explanation for the fewer adverse outcomes in our stroke patients with BD: under-diagnosis of adverse events and inadequate treatment of those conditions. However, the adverse events studied (i.e. in-hospital mortality, acute respiratory failure, and use of mechanical ventilation) are high severity conditions and not readily obscured. Furthermore, the hospitalization variables, length of stay and costs did not differ between stroke patients with BD and those without BD. Taken together, it is less likely that inequities in standard stroke care occurred among our study patients with BD. Recent systemic reviews and large-scale meta-analysis have suggested that antipsychotics and antidepressants generally increase the risk of stroke [27,28]. Further researches would be of interest to examine the effect of medications on in-hospital mortality and adverse event rates in stroke patients with BD.

The study provided a sufficient sample to investigate the outcomes of interest. Use of propensity score adjustment to improve the statistical estimation of risks mitigates selection bias. Nevertheless, several methodological limitations should be acknowledged in interpreting the results. First, diagnostic validity in claims data is often questioned. In the present study, the diagnosis of BD was based on the ICD code. No information from psychiatric evaluations was available. Although the National Health Insurance Bureau randomly reviews medical records to verify the diagnoses and accuracy of coding, our epidemiological research may still exist limitation of under-diagnosis as mentioned previously in the literature. The prevalence of BD was only about 0.2% among the adult patients with stroke in this study. Second, our data lack information on the severity of BD. One potential bias is mortality censoring of patients with BD. More severe patients with BD may be censored due to premature death from other causes such as suicide or other medical conditions. Third, direct measurements of stroke severity are not available in NHIRD database. We used proxy variables (e.g. length of hospital stay and hospitalization costs) which are documented to be correlated with stroke severity measures, such as National Institute of Health Stroke Scale [29–31]. Fourth, lifestyle variables such as cigarette smoking, alcohol consumption, dietary habit, and physical activity are not available in this database. Unhealthy lifestyle behaviors usually have adverse implications for stroke outcomes [10]. Given the direction of findings, life-style factors are less likely to be the confounders. Fifth, our findings may not generalized to other ethnic populations given that differences in stroke characteristics by ethnicity have been reported in the literature [8,32]. We only included hospitalized patients with BD in the study. As previous studies shown, some patients with BD with stroke may be neglected or not brought to medical establishments, who would be excluded from study. Their outcomes may be worse than those of our study sample. Sixth, we selected adverse outcomes associated with significant morbidity and mortality. The functional status following the stroke remains unknown in our sample. Patients with BD with stroke may suffer higher level of disability due to the double disability and worth further investigation.

### Conclusions

We found that with the comparable hospitalization costs and length of stay, stroke patients with BD had lower in-hospital mortality and adverse events rates compared to control patients in this population based study in Taiwan. Further studies are needed to investigate the mediating mechanisms for the observed findings.
